# Microwaves as a novel seed priming method to augment salinity tolerance via regulating the physiological homeostasis and oxidative defense modes in *Gypsophila paniculata* plants

**DOI:** 10.1186/s12870-025-07762-6

**Published:** 2025-12-03

**Authors:** Mahmoud A. El-Ashwah, Magdy A. Barsoom, Hani S. Saudy, Warda A. Aly, Ayman K. Ibrahim

**Affiliations:** 1 Ornamental Plants and Landscape Gardening Res. Dept., Hort. Res. Inst., ARC, Giza, Egypt; 2 Agronomy Department, Faculty of Agriculture, Ain Shams University, Cairo, 11566 Egypt; 3 Department of Horticulture, Faculty of Agriculture, Ain Shams University, Cairo, 11566 Egypt

**Keywords:** Flower quality, Microwaved *Gypsophila paniculata* seeds, Ornamentals, Osmoregulation, Salt injuries

## Abstract

**Purpose:**

The physical seed priming methods are becoming promising due to their lower application costs and greater safety compared with the chemical methods. Although many benefits of microwaves as a physical treatment have been proven for humans and animals, there are not enough studies to highlight their importance for plants, especially under stressful conditions such as salinity. Therefore, possibility of using microwaves to enhance salt tolerance of *Gypsophila paniculata* plants was investigated.

**Methods:**

Seeds of *G. paniculata* were exposed to microwaves for three exposure times (5, 10 and 15 s), in addition to the control treatment (without microwave treatment). Seeds were then sown in pots and the produced plants irrigated with saline water at salinity concentrations of 0, 1000, 2000 and 4000 mg L^− 1^. Growth parameters, flowering behavior, plant pigments, nutrients content and oxidative stress indices were estimated.

**Results:**

Raising concentration of salts in the irrigation water resulted in considerable declines in *G. paniculata* vegetative growth and flowering traits, leaf nutritional status, plant pigments, and carbohydrates percentage while proline, catalase activity and malondialdehyde increased. Plants produced from microwaved seeds for 15 s exhibited the maximum increases of vegetative growth and flowering traits, leaf nutrients content, plant pigments, carbohydrates percentage, proline, and catalase activity with significant reduction in malondialdehyde. Under different salinity levels, microwaving seeds for 15 s was the efficient treatment for improving growth, flower quality, nutrients homeostasis, biochemical constituents and oxidative stress indices *G. paniculata* plants.

**Conclusion:**

The sensitivity of *G. paniculata* to salinity was reduced by applying microwave technology, as the physiological balance was regulated, thus improving the growth and flowering features. Microwave influences may contribute in the metabolism and biosynthesis of osmolytes and antioxidant defensive modes under salt stressful. Accordingly, sowing the microwaved seeds for 15-second as a novel seed priming could be practiced to enhance salt tolerance of *G. paniculata* crop grown in arid regions.

**Supplementary Information:**

The online version contains supplementary material available at 10.1186/s12870-025-07762-6.

## Introduction

The ornamental plants have recently received great attention in scientific studies due to their great importance as a source of high income for farmers in hot regions [1–4]. *Gypsophila paniculata* (My Pink) is a popular cut flower plant utilized as ornamental and therapeutic purposes [5]. Due to its aesthetic value, *G. paniculata* is grown both as a garden plant and for floral arrangements [6]. It is a tiny perennial herbaceous shrub belonging to the Caryophyllaceae family of blooming plants, it may grow to a height of one meter and a width of one meter [7, 8]. Although it is native to Eastern Europe, it can be grown anywhere in the globe, in fields or in greenhouses. It is produced as a cut flower for use in floral arrangements in vases and bouquets or for gardens [9]. *G. paniculata* is easily distinguished by its rosette of grayish-green basal leaves and linear lanceolate leaves, from which the flower stems emerge and end in masses of branching stems covered with clouds of small white or pink flowers [10]. The growth environment, notably day length and temperature, has a significant impact on how *G. paniculata* blooms [11]. It has been observed that *G. paniculata* forms a rosette in environments with limited sunlight and low temperatures [12]. However, edaphic challenges, especially increasing salt concentrations, restrict the expansion of *G. paniculata* cultivation in arid ecosystems.

The economic crops in arid ecosystems are often subjected to different eco-stresses, resulting in depression in quantitative and qualitative characteristics [13–16]. In this concern, salinity as an abiotic stressor is widely spread in arid and semiarid zones causing remarkable losses in agricultural production [17–19]. Due to osmotic stress inducement and excess accumulation of sodium (Na^+^) and chloride (Cl^−^) ions in plant cells by salinity, turmoil in ionic homeostasis with toxicity were noticed [20–23]. All vital plant processes such as photosynthesis and cellular metabolism detrimentally influenced by salinity due to impacts of osmotic stress and ionic toxicity [24–27]. Further, high salinity affects the absorption of nutrients that the plant needs [28–32]. Consequently, chlorophyll injured and plant growth retarded under the impact of salinity, thus crop yield and quality declined [33, 34].

To overcome the issues of salinity impacts on plant crops with production sustainability, pre-planting treatments could be considered a substantial solution [35–37]. In this regard, microwave radiation as a seed priming method is one of the most famous physical treatments [38]. Microwave is thought, in particular to be the most essential physical therapies for pre-sowing seed treatments [39]. Non-ionizing radiation like microwaves possesses lower energy and frequency, while having a longer wavelength. It does not produce charged ions when it moves through the object, yet it is adequate to elevate an electron to a higher energy level and induce biological effects, which are primarily thermal, non-invasive, and harmless to the plants [40]. The duration and frequency of exposure to microwave radiation are the factors affecting germination and growth. Low exposure to microwave radiation has a positive effect on accelerating seed germination and improving growth. However, long exposure may reduce plant growth [41]. Microwaves have been found to cause changes in membrane of cells permeability and cell growth rate, in addition to interference with ions and organic compounds such as proteins [42].

Despite the beneficial action of microwave on growth of some plants, there is no available knowledge on the response of *G. paniculata* as a valuable ornamental plant to treatment with microwave at different exposure times. The present work hypothesized that microwaves use as a seed treatment might motivate the tolerance of *G. paniculata* plants against salt stress. Therefore, seeds of *G. paniculata* were subjected different exposure times of microwaves, and grown under saline irrigation water with different salt concentrations to assess changes in growth, flowering and oxidative stress indices.

## Materials and methods

At the experimental farm of ornamental plants and landscape gardening, Hort. Res. Inst., Giza, Egypt, ARC, a pot experiment was carried out in open field circumstances during 2021-22 and 2022-23 seasons with the aim of improving the ability of *Gypsophila paniculata* to tolerate irrigation water salinity using microwave radiation. The ambient conditions of the experimental site were characterized by an average temperature of 23 °C and a relative humidity of 62%.

The experiment involved 4 × 4 factorial levels arranged in a complete randomized design using 4 replications, each replicate involved 5 pots (5 biological replicates) for each level. In each season, seeds of *G. paniculata* were obtained from Department of Ornamental Plants and Landscape Gardening Research, Horticulture Research Institute, agricultural Research Centre, Giza, Egypt. Before sowing, the seeds were exposed to microwave radiation using a home oven (output at a frequency of 2450 MHz, Model Mo6T, single phase, 220 V., 50 Hs., 1.3 kW). In addition to the untreated seeds group (check treatment), other three groups of seeds were subjected to microwave radiation for 5, 10 and 15 s. The seeds were sown and irrigated by tap water immediately after microwave treatment in the nursery on the 10th September in both seasons. After 21 days, on the first of October, one healthy seedling with lengths of 10 ± 1 cm was planted in plastic pots having diameter of 30-cm and containing a loamy soil. Table 1 shows the physical and chemical properties of the soil used. A week after sowing, the plants were irrigated with four levels of saline water containing sodium chloride (tap water, 1000, 2000 and 4000 mg L^− 1^, equalizing EC of 0.63, 1.56, 3.12 and 6.25 dS m^− 1^, respectively), twice a week with three-day interval using solution volume of 500 ml per pot.

**Table 1:** The physical and chemical analyses of the used soil

Soil type	Soil particles (%)			SP	EC (dS m^− 1^)	pH	Cations (meq L^− 1^)					Anions (meq L^− 1^)		
	Sand	Silt	Clay				Ca^++^	Mg^++^	Na^+^	K^+^		HCO_3_	Cl^−^	SO_4_^−−^
Loamy	48.0	35.5	16.5	44.0	1.36	8.28	3.5	2.5	6.63	0.65		0.5	7.5	5.28

### Data recorded

#### Vegetative and floral growth

The plants began flowering in mid-February and continued to produce flowers until the end of the growing season in early March. In this respect, days to flowering was estimated. Next, at early of March, plant height, leaves number plant^− 1^, stem diameter, branches number plant^− 1^, leaves fresh and dry weights, flowers number plant^− 1^ and flower diameter were measured.

#### Nutrients in leaves

In dry leaves, nitrogen % was analyzed using the micro-Kjeldahle method [43]. Phosphorus % was measured colorimetrically using the method described by Cottenie et al. [44]. Potassium % was assessed using a flame photometer configured [43].

#### Biochemical constituents

Photosynthetic pigments, as total chlorophyll, in fresh leaves were estimated [45]. Anthocyanins in flowers were quantified following the procedure outlined by Saric et al. [46]. Total carbohydrates based on dry matter were estimated according to Dubois et al. [47]. Free proline in dry leaves was determined according to Bates et al. [48]. The content of malondialdehyde (MDA) in fresh leaves was measured according to Du and Bramlage [49]. Catalase (CAT) activity was assayed in fresh leaves according to Jiang and Zhang [50].

### Statistical analysis

All data recorded were statistically analyzed using the method of 2-way analysis of variance (ANOVA) for a complete randomized design in a factorial experiment as outlined by Gomez and Gomez [51] utilizing the “MSTAT-C” computer software package [52]. The means of all treatments were assessed using Duncan’s multiple range tests at a 5% level of probability [53].

## Results

### Vegetative growth

Raising the salinity level in the irrigation water resulted in considerable declines in vegetative growth traits of *G. paniculata* reaching the minimum depression at salinity of 4000 mg L^− 1^ (Table 2). The reductions in growth traits due to the salinity level of 4000 mg L^− 1^ amounted to 37.2 and 37.9% for plant height, 28.8 and 25.0% for stem diameter, 36.8 and 37.0% for branches number plant^− 1^, 36.1 and 34.3% for leaves number plant^− 1^, 56.3 and 53.0% for leaves fresh weight plant^− 1^ and 69.7 and 68.4 for leaves dry weight plant^− 1^ in 2021-22 and 2022-23 seasons, respectively.

**Table 2:** Vegetative growth traits of gypsophila paniculata plant as influenced by salinity level and microwave exposure time in 2021-22 (S1) and 2022-23 (S2) growing seasons

Variable		Plant height (cm)		Stem diameter (cm)		Branches number plant^− 1^		Leaves number plant^− 1^		Leaves weight plant^− 1^ (g)			
										Fresh		Dry	
		S1	S2	S1	S2	S1	S2	S1	S2	S1	S2	S1	S2
Salinity, S (mg L^− 1^)													
0		72.3a	78.4a	0.59a	0.64a	24.2a	26.2a	51.8a	54.5a	3.43a	3.79a	0.89a	0.98a
1000		63.2b	65.0b	0.52b	0.56b	19.5b	21.8b	45.0b	47.5b	2.88b	3.23b	0.75b	0.82b
2000		50.7c	54.8c	0.44c	0.49c	18.0c	19.2c	38.3c	40.8c	2.38c	2.73c	0.63c	0.68c
4000		45.4d	48.7d	0.42d	0.48d	15.3d	16.5d	33.1d	35.8d	1.50d	1.78d	0.27d	0.31c
Microwave, M (second)													
0		51.1d	53.3d	0.44c	0.48d	14.7d	15.4d	36.3d	35.6d	2.23d	2.55d	0.56d	0.58c
5		53.2c	57.8c	0.46c	0.51c	16.8c	17.7c	40.9c	42.8c	2.38c	2.71c	0.60c	0.69b
10		60.9b	65.6b	0.50b	0.55b	20.3b	22.1b	43.1b	46.7b	2.62b	2.99b	0.64b	0.69b
15		66.5a	70.2a	0.57a	0.63a	25.3a	28.4a	47.8a	50.5a	2.97a	3.28a	0.73a	0.81a
S×M													
0	0	67.7 cd	72.0c	0.53 cd	0.56d	18.7ef	19.3f	49.3c	51.0 cd	3.13d	3.45 cd	0.80d	0.82c
	5	67.0d	74.0c	0.56c	0.62c	23.3c	24.7d	50.7bc	50.0d	3.22 cd	3.50 cd	0.88b	0.97b
	10	73.0b	79.7b	0.60b	0.68b	24.0bc	26.3c	50.3bc	55.3b	3.52b	3.98b	0.91b	0.96b
	15	81.7a	88.0a	0.66a	0.72a	30. 7a	34.3a	57.0a	61.7a	3.86a	4.24a	0.97a	1.06a
1000	0	55.2f	55.7 fg	0.49ef	0.53e	15.7gh	16.3 g	40.3f	42.7f	2.48 h	2.87 g	0.69f	0.73d
	5	58.8e	60.3de	0.50de	0.55d	17.0 fg	19.3f	42.7e	45.3e	2.71 g	3.05f	0.71f	0.82c
	10	68.8 cd	71.7c	0.52 cd	0.55d	20.0de	21.7e	45.0d	49.3d	3.00e	3.41d	0.75e	0.81c
	15	70.0c	72.3c	0.55c	0.60c	25.3b	29.7b	51.8b	52.7c	3.33c	3.58c	0.84c	0.93b
2000	0	44.2 h	46.3 h	0.39hi	0.44 g	13.3ij	14.7 h	32.0 h	33.3i	2.07i	2.45 h	0.53 h	0.53f
	5	45.8 h	52.3 g	0.40hi	0.45 g	14.7hi	15.3gh	37.7 g	39.7 g	2.17i	2.46 h	0.60 g	0.69de
	10	51.7 g	57.3ef	0.46 fg	0.52e	20.0de	21.3e	40.7f	45.0e	2.44 h	2.97 g	0.62 g	0.67e
	15	61.2e	63.0d	0.51de	0.57d	24.0bc	25.3 cd	42.7e	45.0e	2.85f	3.21e	0.75e	0.84c
4000	0	37.3j	39.0i	0.35j	0.39 h	11.0k	11.3i	23.7i	27.3j	1.24i	1.41k	0.21k	0.24i
	5	41.0i	44.7 h	0.37ijj	0.43 g	12.0jk	11.3i	32.7 h	36.3 h	1.40k	1.82j	0.23k	0.26i
	10	50.2 g	53.7 g	0.42gh	0.47f	17.0 fg	19.0f	36.3 g	37.0 h	1.53k	1.79j	0.27j	0.32 h
	15	53.0 fg	57.3ef	0.54 cd	0.62c	21.3d	24.3d	39.7f	42.7f	1.85j	2.10i	0.36i	0.42 g

According to Duncan’s multiple range test at *p*˂0.05, different letters indicate significant differences between means for each factor levels in each column

Data listed in Table 2 show that the effect of different exposure times to microwave radiation had clear positive influence with increasing exposure time, as plant growth traits reached the maximum values when seeds exposed to 15 s. In the first season, microwave exposure time for 15 s achieved the greatest values of plant height (30.1%), stem diameter (29.5%), branches number plant^− 1^ (72.1%), leaves number plant^− 1^ (31.7%), leaves fresh weight plant^− 1^ (33.2%) and leaves dry weight plant^− 1^ (30.4%), as compared to the control treatment. The corresponding increases in the second season were 31.7, 31.2, 84.4, 41.9, 28.6 and 39.4%, respectively.

As for the interaction between salinity level and microwave exposure time, favorable effect of microwave on growth traits of *G. paniculata* with increasing the exposure time under different salinity levels (Table 2). Under the control treatment of salinity (0 mg L^− 1^), microwave exposure time for 15 s was the potent treatment, surpassing the other microwave treatments for enhancing all growth traits of *G. paniculata*. Also, microwave exposure time for 15 s showed the most improvements in all growth traits under different salinity levels, equaling microwave exposure time for 10 s for plant height under salinity of 1000 mg L^− 1^ (in both seasons) and 4000 mg L^− 1^ (in the first season), for stem diameter under salinity of 1000 mg L^− 1^ (in the first season) and for leaves number plant^− 1^ under salinity of 2000 mg L^− 1^ (in the second season).

### Floral growth

Flowering status of *G. paniculata* dramatically changed with variation in salt concentration of irrigation water (Table 3). Increasing salt level in irrigation water resulted in a gradual significant delay in flowering date (days to flowering) by 7.5 and 9.6 days, 11.2 and 12.6 days, and 25.6 and 26.4, days with the concentrations of 1000, 2000 and 4000 mg L^− 1^ in the first and second seasons, respectively, compared to the control group. Further, flower diameter and flowers number plant^− 1^ decreased as salinity level increased. Owing to irrigation by water having salinity of 1000, 2000 and 4000 mg L^− 1^, reductions in flower diameter and flowers number plant^− 1^ amounted to 14.3 and 17.6%, 22.6 and 17.2%, and 37.2 and 40.5%, in the first season, as well as 11.4 and 17.8%, 20.6 and 19.6%, and 35.1 and 39.6%, in the second season.

**Table 3:** Flowering attributes of gypsophila paniculata plant as influenced by salinity level and microwave exposure time in 2021-22 (S1) and 2022-23 (S2) growing seasons

Variable		Days to flowering		Flower diameter (cm)		Flowers number plant^− 1^	
		S1	S2	S1	S2	S1	S2
Salinity, S (mg L^− 1^)							
0		109.8d	105.2d	2.17a	2.28a	39.3a	44.5a
1000		117.3c	114.8c	1.86b	2.02b	32.4b	36.6b
2000		121.0b	117.8b	1.68c	1.81c	28.6c	32.8c
4000		135.4a	131.6a	1.34d	1.48d	23.4d	26.9d
Microwave, M (second)							
0		121.8a	118.3a	1.57d	1.70d	26.0d	30.6c
5		121.0b	117.7a	1.62c	1.78c	27.0c	31.6c
10		120.6bc	116.7b	1.86b	1.97b	32.5b	36.9b
15		120.3c	116.6b	2.17a	2.15a	38.3a	41.7a
S×M							
0	0	111.0f	106.0f	1.90d	2.00de	32.3de	37.7 cd
	5	109.7 fg	105.0f	2.00c	2.23c	35.0c	40.0c
	10	109.3 g	104.3f	2.20b	2.37b	41.3b	45.7b
	15	109.3 g	105.3f	2.37a	2.53a	48.7a	54.7a
1000	0	117.7e	115.3cde	1.67e	1.83 fg	26.3 g	31.0e
	5	117.3e	115.0cde	1.73e	1.90ef	26.7 g	31.7e
	10	117.7e	114.7de	1.87d	2.10d	35.0c	40.0c
	15	116.7e	114.0e	2.17b	2.27bc	41.7b	43.7b
2000	0	121.7c	119.7b	1.53f	1.67 h	25.3 g	30.3e
	5	121.7c	118.7b	1.53f	1.63hi	25.0 g	29.0ef
	10	120.7 cd	116.0c	1.73e	1.87f	30.7ef	35.0d
	15	120.0d	116.7d	1.93 cd	2.07d	33.3 cd	36.7d
4000	0	136.7a	132.3a	1.17 h	1.30j	20.0i	23.0 h
	5	135.3ab	132.0a	1.20 h	1.33j	21.3hi	25.7gh
	10	134.7b	131.0a	1.37 g	1.53i	23.0 h	27.0 fg
	15	135.0b	130.7a	1.63e	1.73gh	29.3f	31.7e

According to Duncan’s multiple range test at *p*˂0.05, different letters indicate significant differences between means for each factor levels in each column

Days to flowering of plants produced from microwave-treated seeds for 10–15 s reduced compared to microwave-untreated seeds (Table 3). All tested microwave exposure times surpassed the control treatment for enhancing flower diameter and flowers number plant^− 1^ in both seasons. However, microwave exposure time for 15 s was the effective treatment for increasing flower diameter and flowers number plant^− 1^, outperforming the other exposure times in both seasons.

As for the interaction, the longest days to flowering were observed under irrigation with water containing 4000 mg L^− 1^ salts × microwave exposure time of 10 and 15 s in the first season and microwave exposure time of 0, 5, 10 and 15 s in the second season (Table 3). While, the shortest days to flowering were recorded under control treatment (0 mg L^− 1^) × microwave exposure time of 5, 10 and 15 s in the first season and microwave exposure time of 0, 5, 10 and 15 s in the second season. At no salt stress (0 mg L^− 1^), microwave exposure time of 15 s possessed the maximal increases in flower diameter and flowers number plant^− 1^ in both seasons. Under salt stress (2000 or 4000 0 mg L^− 1^), seeds microwaved for 15 s produced plants bearing flowers with the largest diameter and number in both seasons. Microwaved seeds for 10 s came in the second order surpassing the control treatments in this respect. Under the three levels of salinity stress, the differences in flower diameter and flowers number plant^− 1^ between the microwaved seeds for 5 s and non-microwaved seeds treatment were not significant in both seasons.

### Nutrients in leaves

The presented data in Table 4 showed that as the salinity level increased, the leaf nutrients content decreased. Therefore, the lowest values of N, P and K in both seasons were obtained with 4000 mg L^− 1^, except for N in the first season where the lowest values was observed with 2000 mg L^− 1^. Microwave radiation increased N, P and K accumulation in the leaves, reaching the highest level at 15 s of exposure time in both seasons (Table 4).

**Table 4:** Leaf nutrients content of gypsophila paniculata plant as influenced by salinity level and microwave exposure time in 2021-22 (S1) and 2022-23 (S2) growing seasons

Variable		Nitrogen (%)		Phosphorus (%)		Potassium (%)	
		S1	S2	S1	S2	S1	S2
Salinity, S (mg L^− 1^)							
0		1.72a	2.11a	0.91a	0.83a	1.60a	1.62a
1000		1.64b	1.77b	0.85b	0.77b	1.56b	1.46b
2000		1.58d	1.60c	0.72c	0.64c	1.49c	1.34c
4000		1.47c	1.30d	0.64d	0.56d	1.46c	1.08d
Microwave, M (second)							
0		1.58c	1.34d	0.68d	0.60d	1.49c	1.16d
5		1.58c	1.39c	0.71c	0.63c	1.50c	1.26c
10		1.62b	1.73b	0.79b	0.71b	1.55b	1.46b
15		1.63a	2.32a	0.94a	0.86a	1.57a	1.61a
S×M							
0	0	1.69b	1.61 g	0.80ef	0.72ef	1.57d	1.47de
	5	1.68b	1.66f	0.81def	0.73def	1.58c	1.50d
	10	1.75a	2.75a	0.89c	1.05a	1.62a	1.79a
	15	1.75a	2.42c	1.13a	0.81c	1.62a	1.70b
1000	0	1.61e	1.42i	0.77 fg	0.69 fg	1.52ef	1.28 g
	5	1.63d	1.44i	0.83de	0.75de	1.53e	1.30 g
	10	1.64 cd	2.52b	0.85 cd	0.88b	1.59c	1.71b
	15	1.66c	1.69f	0.96b	0.77 cd	1.6b	1.56c
2000	0	1.55 g	1.30j	0.77 fg	0.54i	1.47 h	1.04i
	5	1.56 g	1.45i	0.83de	0.54i	1.46 h	1.31 g
	10	1.58f	2.12d	0.85 cd	0.80c	1.51f	1.57c
	15	1.61e	1.53 h	0.96b	0.63 g	1.52ef	1.42ef
4000	0	1.45j	1.01k	0.53j	0.45j	1.41i	0.84k
	5	1.46j	1.02k	0.57j	0.48j	1.41i	0.92j
	10	1.48i	1.88e	0.69 h	0.73ef	1.48 g	1.39f
	15	1.50 h	1.30j	0.81def	0.61 h	1.52ef	1.16 h

According to Duncan’s multiple range test at *p*˂0.05, different letters indicate significant differences between means for each factor levels in each column

There was fluctuation in leaf nutrients content between the microwave exposure times of 10 and 15 s under different salinity levels in both seasons. Generally, the superiority was observed for 15 s in the first season and for 10 s in the second seasons. However, in the first season, there were no significant differences between 10 and 15 s for N under 0 or 1000 mg L^− 1^ and K under 0 or 2000 mg L^− 1^.

### Biochemical constituents

**Fig. 1:**
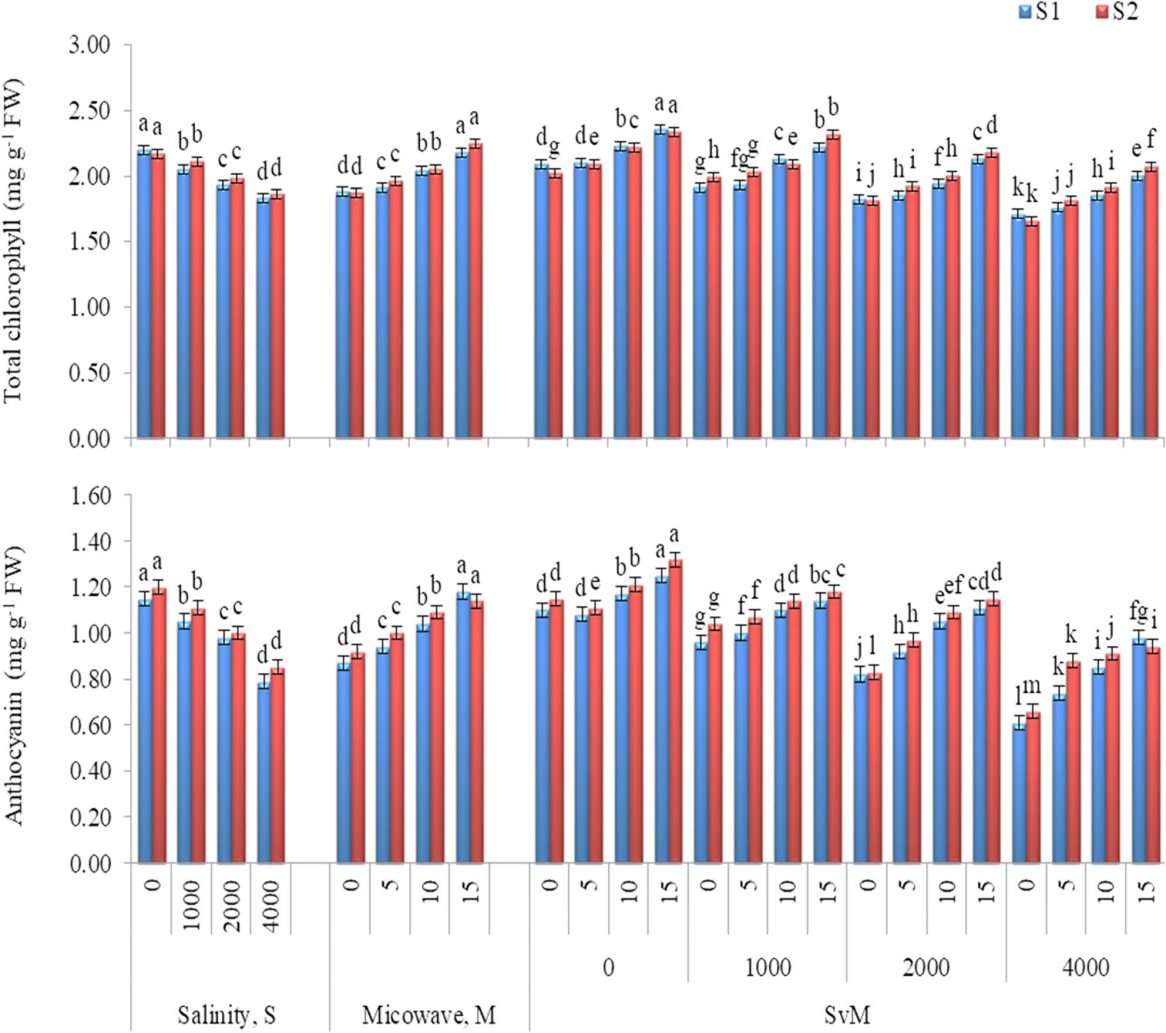
Total chlorophyll and anthocyanin of *Gypsophila paniculata* plant as influenced by salinity level and microwave exposure time in 2021-22 (S1) and 2022-23 (S2) growing seasons. Error bars represent ± SE. According to Duncan’s multiple range test at *p*˂0.05, different letters indicate significant differences between means for each factor levels

**Fig. 2:**
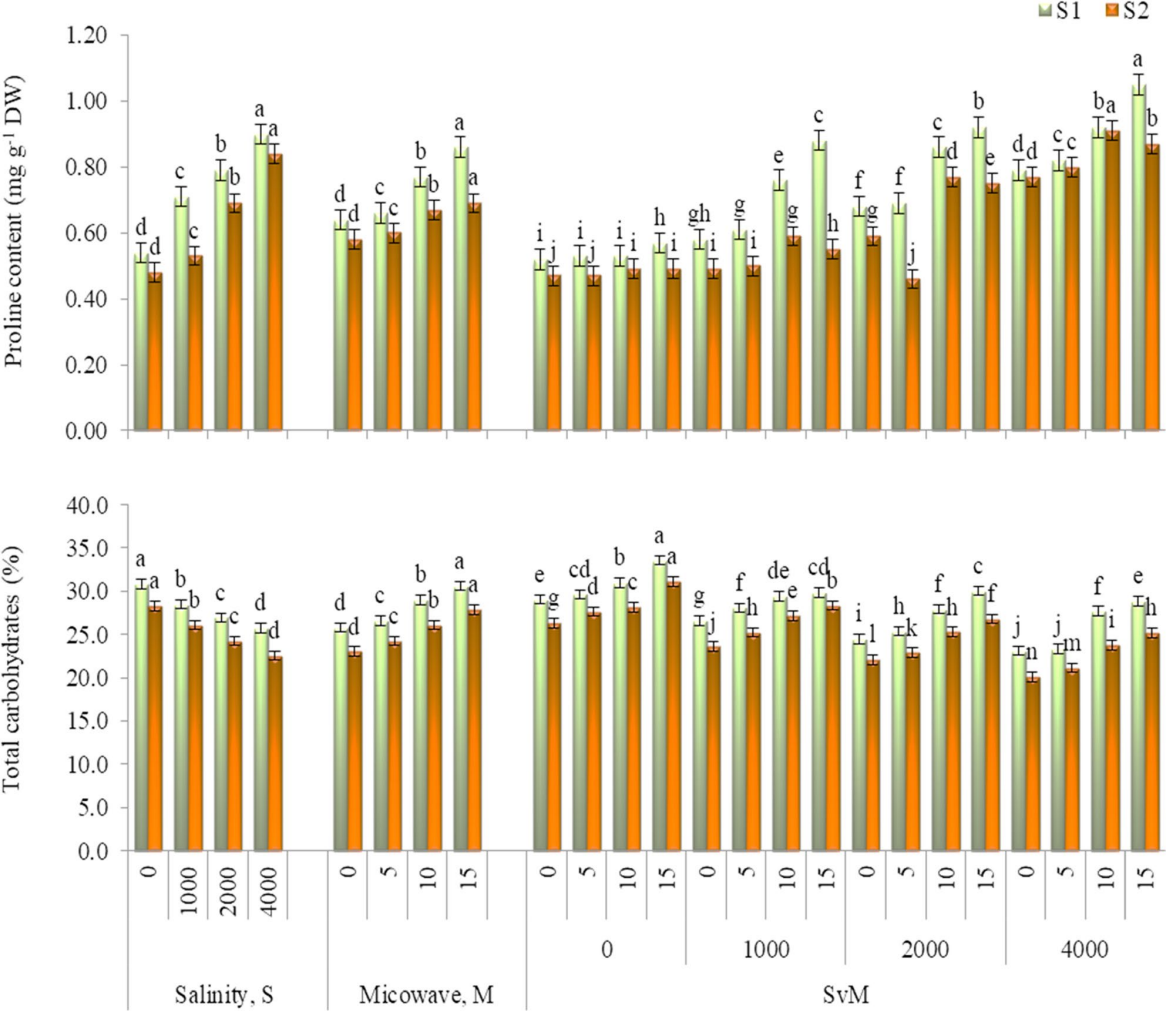
Proline content and carbohydrates% of *Gypsophila paniculata* plant as influenced by salinity level and microwave exposure time in 202-/22 (S1) and 2022-23 (S2) growing seasons. Error bars represent ± SE. According to Duncan’s multiple range test at *p*˂0.05, different letters indicate significant differences between means for each factor levels

Total chlorophyll and anthocyanin (Fig. 1), and proline and total carbohydrates (Fig. 2) significantly influenced by salinity level and microwave radiation and their interaction in both seasons. Total chlorophyll, anthocyanin and total carbohydrates decreased and proline increased as salinity level increased. Herein, the maximum values of total chlorophyll, anthocyanin and total carbohydrates were recorded with the control treatment (0 mg L^− 1^), while the lowest values were obtained with 4000 mg L^− 1^. Contrariwise, the maximum accumulation of proline was obtained with the salinity level of 4000 mg L^− 1^.

Microwave application for 15 s possessed the maximum increases in total chlorophyll, anthocyanin, proline and total carbohydrates in both seasons, outperforming the control treatment by 1.15 and 1.20 folds, 1.35 and 1.23 folds, 1.34 and 1.18 folds and 1.18 and 1.21 folds in the first and second seasons, respectively.

As for the interaction, generally, application of microwave for 15 s under non-salt stress achieved the highest increases in total chlorophyll, anthocyanin, and total carbohydrates in both seasons. While, the maximal increases in proline were observed under salinity level of 4000 mg L^− 1^ with microwave for 15 s in the first season and with microwave for 10 s in the second season. Furthermore, under different salinity levels (1000, 2000 or 4000 mg L^− 1^) microwave for 15 s was the remarkable treatment for enhancing total chlorophyll, anthocyanin, and total carbohydrates in both seasons, statistically equaling microwave for 10 s treatment in total carbohydrates in the first season..

**Fig. 3:**
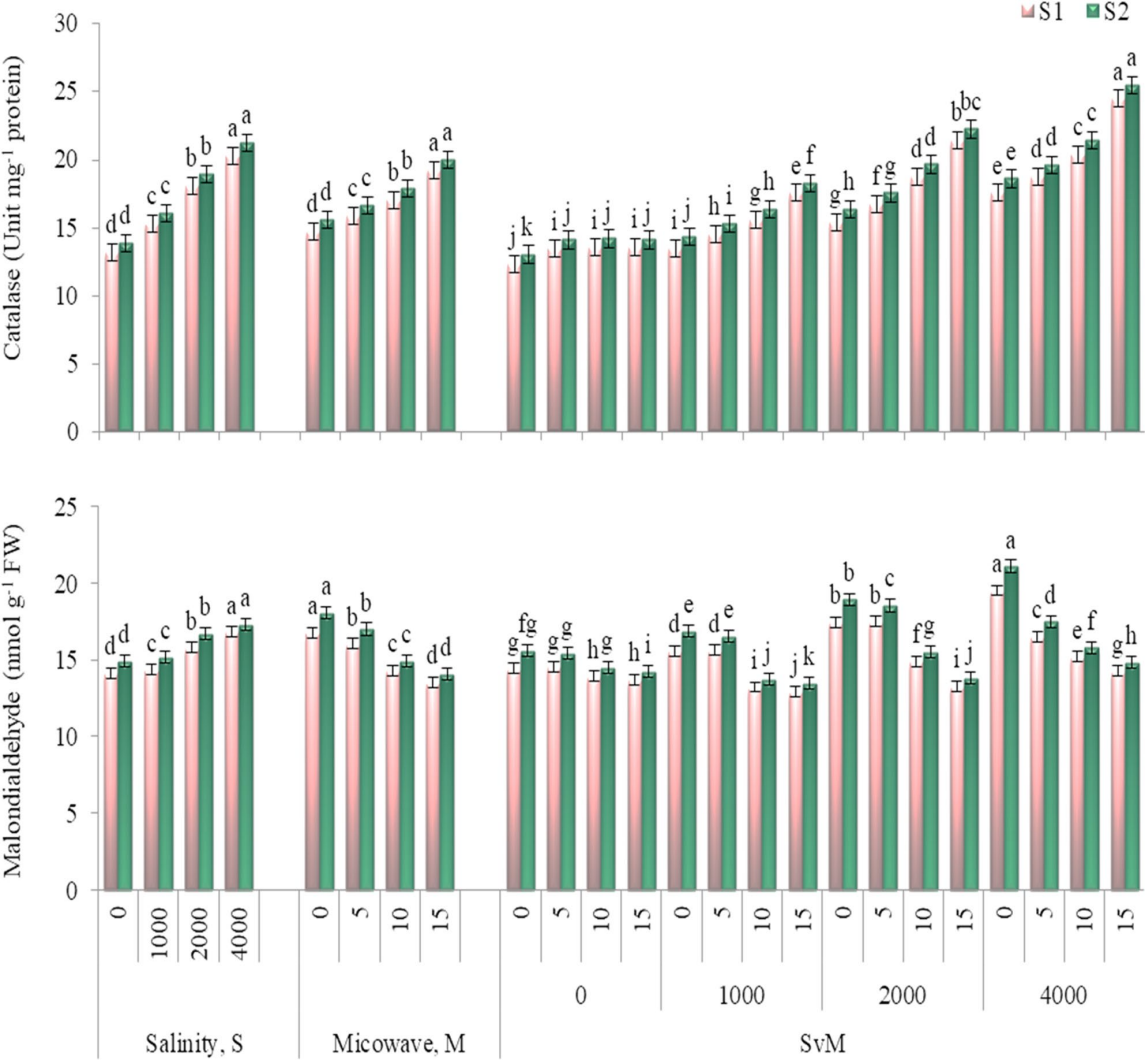
Catalase activity and malondialdehyde content of *Gypsophila paniculata* plant as influenced by salinity level and microwave exposure time in 2021-22 (S1) and 2022-23 (S2) growing seasons. Error bars represent ± SE. According to Duncan’s multiple range test at *p*˂0.05, different letters indicate significant differences between means for each factor levels

Concerning the oxidative stress indices, it has been observed the increased levels of catalase activity and MDA content as salinity level elevated (Fig. 3). Thus, salinity of 4000 mg L^− 1^ exhibited the greatest values surpassing the control treatment by 53.4 and 53.3% for catalase activity and 18.9 and 61.0% for MDA content in the first and second seasons, respectively.

Application of microwave for 15 s was the potent treatment for stimulating the oxidative stress indices, increasing catalase activity by 31.0 and 28.5%, with decreasing MDA content by 19.2 and 22.2% as compared to the control treatment (without microwave treatment) in the first and second seasons, respectively.

Under salinity of 4000 mg L^− 1^, plants produced from microwaved seeds for 150 s had the highest catalase activity and the lowest MDA content in both seasons.

## Discussion

Under various circumstances, plant growers are compelled to irrigate their crops by saline water. In this case, the cultivated plants are exposed to salt injury that may lead to reduced growth and marketable yield. It gets worse with ornamental plants that are so sensitive to salinity such as *Gypsophila*: Salinity suppresses root growth [26] via reducing cell division and interruption of cell membrane [54]. Owing to reduced potassium while sodium and chloride increased under salinity, an ionic imbalance has occurred. Therefore, salinity restricted the uptake of nutrients necessary for plant growth [55]. When newly established *Gypsophila* were exposed to increasing concentrations of NaCl, plant height, leaf number, fresh and dry weight of shoot and root systems, leaf chlorophyll content, floral characteristics progressively decreased while proline content increased [56, 57]. Treating *Gypsophila aucheri* with 300 mM NaCl had a detrimental effect on overall plant growth, membrane integrity, and increased oxidative stress [58]. Under salt stress conditions, significant vegetative and floral growth traits of calendula plants, and physiological characteristics such as photosynthetic pigments, relative water content, and leaf mineral were significantly decreased, while sodium and proline concentrations in leaves increased [59]. Due to the severe provocation of ROS formation by the photosynthetic apparatus as a result of salt stress, leaf pigments are rapidly disintegrated and leaf cell death can occur [60]. Xing et al. [61] reported that treatment with concentrations of 100, 150, and 200 mM NaCl increased MDA and oxygen production, leading to damage cell membrane integrity and protein activity.

The present study documented the beneficial role of microwaves on vegetative growth and flowering behavior under both non-stressed and salt stressed conditions. Under different exposure durations in *Delonix regia* and *Albizzia julibrissin*, microwaving seeds for 5 s exhibited the greatest values of growth rate, plant height, number of leaves per plant, leaf dry weight, root length, leaves chlorophyll content [62]. When microwaves were applied to dahlia plants, a direct correlation was observed between chlorophyll levels in the leaves and anthocyanin levels in the flowers with increased microwave energy [63]. The microwave exposure durations for 5 s in *Acacia farnesiana* and for 10 s in *Acacia saligna* achieved the maximum values of growth traits and chemical composition [64]. Concerning the favorable influence of microwave on flowering features, it has been reported that chrysanthemums plants grown from microwaves-treated explants exhibited longer shoots featuring inflorescences with larger diameters and varied phenotypes [65]. Horikoshi et al. [66] demonstrated that the treatment of *Arabidopsis thaliana* by microwaves promoted the flowering induction. Pre-planting exposure of seeds to physical treatments such as microwave radiation results in frequent changes in physio-biochemical processes within the seeds, which can ultimately be translate into production of more vigorous seedlings with increased the final survival rate [67, 68]. In this respect, exposure to microwave has been shown to have simulative effects on chlorophyll and protein synthesis [69]. Microwave treatment resulted in improving photosynthetic pigments and growth biomass [70]. As a result of the internal microwave reactions that lead to the reduction of growth inhibitors (abscisic acid) with the activation of growth stimulants (auxin), significant improvements were recorded in the germination rate, water absorption, seedlings weight and length, in addition to an increase in chlorophyll and carotenoids levels [71, 72].

The different levels of NaCl stress showed a noticeable arousal in proline level, carbohydrates content, lipid peroxidation (MDA) and catalase activity. On the other hands, application of microwaves was efficient for reducing the levels of MDA and elevating proline concentration, carbohydrates % and catalase activity. In this respect, the mode of action of microwave radiation is likely to involve accumulation of proline content and capture of ROS helping in maintaining relative water content and higher osmotic potential in plants leading to improved water use efficiency [73]. The remarkable accumulation of osmo-protectants such as proline and soluble sugars is one of the most significant features that demonstrate the ability of plants to tolerate salt stress. These organic compounds can work together in concert to organize plant water uptake and prevent tissue desiccation by resetting the cellular osmotic potential under unfavorable conditions [74]. The results of the present research showed that microwaves may appear to be beneficial in modifying the redox imbalance by reducing the H_2_O_2_ content as the MDA content decreased with increasing microwave exposure time, thus maintaining the integrity of the cell membrane. This observation elucidates the distinct action of microwaves to reduce the damage of salinity on *Gypsophila*. In this regard, Chen et al. [75] showed that the hazards of ROS generated under salt stress on root and shoot development and growth of wheat seedlings can be dwindled via microwave seed treatment. Therefore, the microwave-treated seeds produced seedlings with longer roots and taller plants than those produced from untreated seeds under salinity stress [76]. In this context, the results of this study indicated that the catalase and MDA increased with increasing salinity level. On the contrary, microwaving *Gypsophila* seeds reduced MDA levels while increased catalase activity. By studying the effect of salinity on three species of *Tagetes patula*, the results indicated a consistent increase in antioxidant compounds, as the enzyme catalase concentration increased with increasing salinity levels [77]. Moreover, in *Portulaca oleracea*, the concentration of MDA increased with increasing salt concentration of irrigation water [78]. While, *Arabidopsis thaliana* plants whose seeds were exposed to microwave and grown in vitro showed lower MDA content in the leaves compared to the control [79]. *Fagopyrum tataricum* seeds were microwaved at different power levels of 200, 400, 600, and 800 W for 10 or 30 s, showing a positive effect on the activity of the catalase enzyme [80]. Also, Bian et al. [81] indicated that the appropriate microwave treatment could effectively improve the antioxidant enzyme activity such as catalase of *Tartary buckwheat* sprouts and enhance their antioxidant capacity.

Accordingly, the current study exhibited enhancements in proline, carbohydrates content and catalase activity while shrinking MDA in plants produced from microwaves-treated seeds implying that microwave influences may contribute in the metabolism and biosynthesis of osmolytes and antioxidant defensive modes under salt stressful.

## Conclusions

It could be deduce that microwaving *Gypsophila paniculata* (My Pink) seeds as a pre-planting treatment resulted in supporting the plants to be more tolerant against salt stress, where photosynthetic pigments, nutrient homeostasis, and various defensive mechanisms were improved. In this respect, exposing *Gypsophila paniculata* seeds to electromagnetic radiation of microwave for 15-second before planting is recommended to improve vegetative and flowering characteristics, and increase salinity tolerance while maintaining plant quality. However, since the highest exposure time of microwave (15-second) outperformed the lower times for most of the tested traits, further study are needed to evaluate other microwave exposure durations with a focus on genetic analysis to support the current findings and discover new potential outcomes.

## Supplementary Information

Supplementary Material 1.

## Data Availability

The datasets used and/or analyzed during the current research are available from the corresponding author upon reasonable request.

## Declarations

“Regarding Experimental research and field studies on plants, we cultivated *Gypsophila paniculata* plants by ourselves.”
