# Supplementary material for: Microwaves as a novel seed priming method to augment salinity tolerance via regulating the physiological homeostasis and oxidative defense modes in Gypsophila paniculata plants
Source: BMC Plant Biol. 2025 Dec 3;25:1694. doi: 10.1186/s12870-025-07762-6 (PMC12690845; doi:10.1186/s12870-025-07762-6)
Supplement: Supplementary file 1 — Supplementary Material 1. [file 12870_2025_7762_MOESM1_ESM.docx]

| Supplementary Table 1. ANOVA analysis of barley traits as affected by salinity and microwave in 2021/22 season | | | | | | | | | | | | | | | |  |  |  |  |
| --- | --- | --- | --- | --- | --- | --- | --- | --- | --- | --- | --- | --- | --- | --- | --- | --- | --- | --- | --- |
| S.O.V. | D.F. | Mean square (M.S.) | | | | | | | | | | | | | |  |  |  |  |
|  |  | Plant height | Stem diameter | Branches number plant^-1^ | Leaves number plant^-1^ | Leaves fresh weight | Leaves dry weight | Days to flowering | Flower diameter | Flowers number plant^-1^ | N% | P% | K% | Total chlorophyll | Anthocyanin | Proline | Carbohydrates | Catalase | Malondialdehyde |
| Total | 47 |  |  |  |  |  |  |  |  |  |  |  |  |  |  |  |  |  |  |
| Microwave (a) | 3 | 604.9*** | 0.04*** | 261.1*** | 272.0*** | 1.25*** | 0.063*** | 5.02*** | 0.52*** | 383.2*** | 0.008*** | 0.17*** | 0.017*** | 0.22*** | 0.143*** | 0.12*** | 57.4*** | 45.58*** | 26.6*** |
| Salinity (b) | 3 | 1780.4*** | 0.07*** | 164.6*** | 796.05*** | 8.03*** | 0.86*** | 1383.7*** | 1.27*** | 539.1*** | 0.13*** | 0.18*** | 0.048*** | 0.29*** | 0.27*** | 0.27*** | 57.38*** | 115.38*** | 14.29*** |
| a × b | 9 | 12.1*** | 0.002** | 3.0* | 15.3*** | 0.01ns | 0.001* | 0.613ns | 0.003ns | 13.17*** | 3.6389e-4** | 0.004*** | 5.8889e-4*** | 0.002*** | 0.006*** | 0.009*** | 2.13*** | 4.17*** | 2.96*** |
| Error | 32 | 2.67 | 4.0625e-4 | 1.23 | 1.23 | 0.006 | 3.9375e-4 | 2.71 | 0.003 | 1.23 | 9.1667e-5 | 5.7917e-4 | 3.3333e-5 | 2.0419e-4 | 3.8542e-4 | 3.1667e-4 | 0.11 | 0.012 | 0.226 |

| Supplementary Table 2. ANOVA analysis of barley traits as affected by salinity and microwave in 2022/23 season | | | | | | | | | | | | | | | |  |  |  |  |
| --- | --- | --- | --- | --- | --- | --- | --- | --- | --- | --- | --- | --- | --- | --- | --- | --- | --- | --- | --- |
| S.O.V. | D.F. | Mean square (M.S.) | | | | | | | | | | | | | |  |  |  |  |
|  |  | Plant height | Stem diameter | Branches number plant^-1^ | Leaves number plant^-1^ | Leaves fresh weight | Leaves dry weight | Days to flowering | Flower diameter | Flowers number plant^-1^ | N% | P% | K% | Total chlorophyll | Anthocyanin | Proline | Carbohydrates | Catalase | Malondialdehyde |
| Total | 47 |  |  |  |  |  |  |  |  |  |  |  |  |  |  |  |  |  |  |
| Microwave (a) | 3 | 692.5*** | 0.05*** | 393.7*** | 313.57*** | 1.26*** | 0.11*** | 9.69ns | 0.49*** | 316.6*** | 2.44*** | 0.17*** | 0.5*** | 0.28*** | 0.117*** | 0.034*** | 54.47*** | 43.91*** | 41.22*** |
| Salinity (b) | 3 | 2034.0*** | 0.07*** | 203.3*** | 792.35*** | 8.74*** | 0.93*** | 1431.7*** | 1.41*** | 562.1*** | 1.35*** | 0.18*** | 0.62*** | 0.23*** | 0.266*** | 0.315*** | 73.24*** | 126.76*** | 16.17*** |
| a × b | 9 | 9.9* | 0.003*** | 6.17*** | 14.0*** | 0.4** | 0.003** | 1.34ns | 0.007ns | 16.5*** | 0.07*** | 0.004*** | 0.012*** | 0.003*** | 0.009*** | 0.003*** | 0.68*** | 4.41*** | 3.45*** |
| Error | 32 | 4.17 | 1.4375e-4 | 0.68 | 1.67 | 0.009 | 7.2292e-4 | 3.38 | 0.004 | 2.9 | 9.3542e-4 | 5.7917e-4 | 0.001 | 1.2917e-4 | 1.9375e-4 | 8.5417e-5 | 0.012 | 0.133 | 0.029 |
